# Authors’ Response to Peer Reviews of “Performance Drift in Machine Learning Models for Cardiac Surgery Risk Prediction: Retrospective Analysis”

**DOI:** 10.2196/60384

**Published:** 2024-06-12

**Authors:** Tim Dong, Shubhra Sinha, Ben Zhai, Daniel Fudulu, Jeremy Chan, Pradeep Narayan, Andy Judge, Massimo Caputo, Arnaldo Dimagli, Umberto Benedetto, Gianni D Angelini

**Affiliations:** 1 Bristol Heart Institute, Translational Health Sciences, University of Bristol, Bristol, United Kingdom; 2 School of Computing Science, Northumbria University, Newcastle upon Tyne, United Kingdom; 3 Department of Cardiac Surgery, Rabindranath Tagore International Institute of Cardiac Sciences, West Bengal, India

**Keywords:** cardiac surgery, artificial intelligence, risk prediction, machine learning, operative mortality, data set drift, performance drift, national data set, adult, data, cardiac, surgery, cardiology, heart, risk, prediction, United Kingdom, mortality, performance, model

*This is the authors’ response to peer-review reports for “Performance Drift in Machine Learning Models for Cardiac Surgery Risk Prediction: Retrospective Analysis.”*

## Round 1 Review

### Anonymous [1]

#### General Comments

*Overall, I think this is a really interesting paper [2]. It is a concept I had never heard of, and I can see very clearly how this is an important consideration. I also think the authors have done excellently to consider a host of different aspects, including feature importance change, beyond the most obvious measurements.*

#### Specific Comments

##### Abstract

*1. “It has been suggested that using Machine Learning (ML) techniques, a branch of Artificial intelligence (AI), may improve the accuracy of risk prediction.” Improve them over what? Specify what the status quo is with regard to first principles and data-driven modeling. This statement is also repeated in the first line of the introduction—what is “conventional” about these models?*

Response: Please note that all line numbers refer to the marked version of the manuscript with tracked changes, uploaded as a supplementary file.

Thank you for this helpful suggestion. This has now been modified in the abstract as follows:

It has been suggested that using Machine Learning (ML) techniques, a branch of Artificial intelligence (AI), may improve the accuracy of risk prediction over traditional mortality risk stratification models.

These traditional scoring methods are generally based on logistic regression with risk factors determined through a consensus across experts within leading cardiac surgery organisations in the United States (STS) or Europe (ES II).

The above important points have been incorporated in lines 31, 103, and 106-108.

*2. “five ML mortality prediction models”—it should be highlighted that these are novel models that you have developed for this paper.*

Response: Thank you for this suggestion. This has now been modified as “Five novel ML mortality prediction models were developed and assessed with EuroSCORE II for relationships…” in lines 39-40.

*3. “geometric average results of all metrics”—it is not all metrics, just the 5 that you have calculated. It is better to just say here “a novel metric called the CEM” or something.*

Response: Thank you for the helpful suggestion. This has been changed to “Performance was assessed using a consensus metric.” in lines 41-44.

##### Introduction

*Why is data set drift a problem? I think you could do more here to highlight how important this is to an audience who might not be dealing with the data themselves and, thus, might not naturally think of examples: for example, changes in treatment guidelines, demographics, new risk factors emerging, or changes in coding practices. You could mention “new” comorbidities such as long COVID.*

Response: Thank you for this interesting comment. We have now included a more extensive explanation of data set drift and its importance in the *Introduction* section.

Introduction

Changes in treatment regimens, demography, new risk factors, adjustments to clinical coding procedures, or the addition of new variables such as the identification of previously unknown conditions such long Covid can all contribute to this phenomenon. The issue of dataset drift is serious, particularly for individuals who depend on the quality or insights of the data but may not analyse it directly. Below are some reasons why this is important:

1. Impact on Decision-Making: Decision-makers may base their choices on erroneous or obsolete information if they rely on drifted or outdated datasets. In the healthcare industry, for example, if changes in treatment recommendations rely solely on historical data, this could result in less-than-ideal patient care because the analysis would not account for newer, more effective therapies.

2. Reduced Model Performance: When dataset drift occurs, machine learning models and predictive algorithms that were trained on historical data may become less accurate or dependable. For instance, a financial prediction model based on antiquated market tendencies may not be able to predict novel market behaviours, which could result in losses.

3. Biased or Inaccurate Insights: Datasets that have drifted may contain biases or errors. Model generalizability may be impacted by changes in demographics, such as adjustments in the age distributions of the population. The prevalence of post-cardiac surgery outcomes may be impacted by newly identified risk factors or circumstances (such as long Covid), necessitating modifications to predictive models in order to preserve accuracy.

4. Challenges in Generalization: Models developed with dated data could have trouble extrapolating to novel scenarios. For instance, Euroscore I was developed in 1999 using 19,030 patients collected over three months (September–December 1995) from 132 cardiac centres in eight countries [13]. Modifications to risk factors over time may have an resulted in its lack of discrimination and calibration compared to its successor score EuroSCORE (ES) II, developed in 2011.

5. Ethical and Fairness Concerns: Drift in the dataset may exacerbate problems with ethics or fairness. A system may reinforce preexisting biases and unfairly target particular groups if it was trained on biased or out-of-date data.

6. Regulatory Compliance: Using historical or drifted data could result in non-compliance with changing standards that need accurate, current information in regulated industries like healthcare.

The above important points have been incorporated in lines 148-181.

##### Methods

*1. Could the same individuals be in both the training and validation set and holdout set, if they had multiple surgeries? If so, this may have introduced some bias into the performance estimates. I do not think you need to redo the analyses, but if you can highlight the degree of overlap, then that would be good. Otherwise, say it was not possible and list it as a limitation.*

Response: Thank you. As National Adult Cardiac Surgery Audit (NACSA) patient identifiers and the Hospital Episode Statistics data set were not available for linkage, it was not possible to determine whether there were any patients in both the training and validation set and holdout set, where they had multiple surgeries. Clinical judgment suggests that the proportion with multiple surgeries would be very low. Nonetheless, future work should consider the collection of such information to minimize any potential bias.

The above important points have been incorporated in lines 644-649.

*2. “As a sensitivity analysis, we excluded the True Negative Rate from the performance evaluation, by calculating the F1 score.” This sentence does not quite make sense to me. The F_1_-score is based on the sensitivity (true negative rate) and the precision (positive predictive value), right? It does not exclude the true negative rate per se; it just does not use it.*

Response: Thank you. As a sensitivity analysis, we calculated the *F*_1_-score, which combines precision and recall without explicitly considering the true negative rate in the performance evaluation.

The above important points have been incorporated in lines 274-277.

Thank you for your expert and invaluable review; this has been really thought provoking and had made significant contributions to improving the journal’s quality of output. Thanks.

We hope this now addresses your queries. Thank you.

### Reviewer CL [3]

#### General Comments

*This manuscript presents an interesting study that explores temporal trends in various performance metrics for different types of prediction models used in the prediction of in-hospital mortality after cardiac surgery in the United Kingdom from 2012 to 2019. The data set was divided into 2 periods: from 2012 to 2016 for model training and internal validation and from 2017 to 2019 for external validation. The study evaluated 5 prediction models: logistic regression, support vector machine (SVM), random forest, extreme gradient boosting (XGBoost), neural network, and European System for Cardiac Operative Risk Evaluation (EuroSCORE) II. The authors aimed to assess the model performance on 5 metrics (1 – expected calibration error [ECE], area under the curve [AUC], 1 – Brier score, F_1_-score, and net benefit) and proposed a composite metric, the clinical effectiveness metric (CEM), calculated as the geometric mean of the 5 mentioned metrics, as the primary metric.*

*The study began with a nontemporal baseline evaluation of different models in the 2017-2019 temporal validation and then conducted a series of drift analyses, including an examination of overall trends from 2012 to 2019, within-period trends in the first 3 months of 2017 and 2019, and between-period trends between the first 3 months of 2017 and 2019. The authors also analyzed drift in variable importance and variable distribution, defined by the temporal change in the ratio of several top-importance features within the data set, to profile data set drift.*

*The authors demonstrated that XGBoost and random forest were the best-performing models, both in nontemporal and temporal evaluations, whereas the EuroSCORE II model exhibited a significant drop in performance. Temporal declines in model performance were observed across all models and were consistent with data set drift.*

*Overall, the question of the generalizability of prediction models, whether temporal or spatial, has long been a topic of discussion in clinical research. This study takes a commendable approach to addressing this question. However, there are some issues that require clarification and revision, including (1) methodological concerns related to the justification of the main metric (CEM) using averaging, and the appropriateness of some statistical tests; (2) the clinical significance of the identified performance drift; and (3) the overall clarity of the study’s design and presentation.*

#### Specific Comments

##### Major Comments

*1. The statement of the study’s objectives should be improved for more clarity, particularly regarding the phrase “verify suspected dataset drift by assessing the relationship between and within performance drift, variable importance drift, and dataset drift across ML and ES II approaches.” It is unclear what is meant by the “relationship between and within.” Does this refer to the analysis of performance drift within and between different periods? The overall study design is quite challenging to grasp initially, even with the graphical overview provided in Figure 1. To enhance clarity, additional details and explanations should be added to the aims, overall design, graphical overview, and text the Methods and Results sections.*

Response: Please note that all line numbers refer to the marked version of the manuscript with tracked changes, uploaded as a supplementary file.

Thank you for this helpful suggestion. We have now improved the *Introduction* and *Methods* sections in terms of making the design and aims easier to comprehend across a wider range of readers.

Introduction

Performance drift in ML is when the performance of the ML models deteriorate over time due to various changes that may reduce the validity of the model’s assumptions at the time of training. The following are the primary reasons for performance drift: (i) Dataset Drift happens when the distribution of the data between the training set and the dataset used for evaluation or prediction varies. For example, if a model is trained on data from one time period but evaluated or used in another time period where the data distribution has changed dramatically, performance may suffer; (ii) Concept Drift occurs when the fundamental relationship between the input features and the target variable shifts over time. The assumptions upon which the model was developed may no longer be valid. In a predictive maintenance model, for example, the behavior of the machinery may alter subtly over time due to numerous causes (such as wear and tear), causing the model to become less accurate as time passes; (iii) Variable Importance Drift: Changes in the significance or importance of various variables/features used by the model to make predictions. Variables that were essential during the model’s training phase may become less important, while other variables may become more influential when the environment or the problem itself evolves. Calibration Drift: Calibration refers to the agreement between expected and actual probabilities of an event; (iv) Calibration drift occurs when the estimated probability of the model grow less dependable over time. This could happen if the model was calibrated based on assumptions about the data distribution that no longer hold true. These different types of drift may also have an interplay effect, and this was shown through a non-cardiac surgery study that used actual dataset drift to verify variable importance detected dataset drift [54].

Changes in treatment regimens, demography, new risk factors, adjustments to clinical coding procedures, or the addition of new variables such as the identification of previously unknown conditions such long Covid can all contribute to this dataset drift phenomenon. The issue of dataset drift is serious, particularly for individuals who depend on the quality or insights of the data but may not analyse it directly. Below are some reasons why this is important:

1. Impact on Decision-Making: Decision-makers may base their choices on erroneous or obsolete information if they rely on drifted or outdated datasets. In the healthcare industry, for example, if changes in treatment recommendations rely solely on historical data, this could result in less-than-ideal patient care because the analysis would not account for newer, more effective therapies.

2. Reduced Model Performance: When dataset drift occurs, machine learning models and predictive algorithms that were trained on historical data may become less accurate or dependable. For instance, a financial prediction model based on antiquated market tendencies may not be able to predict novel market behaviours, which could result in losses.

3. Biased or Inaccurate Insights: Datasets that have drifted may contain biases or errors. Model generalizability may be impacted by changes in demographics, such as adjustments in the age distributions of the population. The prevalence of post-cardiac surgery outcomes may be impacted by newly identified risk factors or circumstances (such as long Covid), necessitating modifications to predictive models in order to preserve accuracy.

4. Challenges in Generalization: Models developed with dated data could have trouble extrapolating to novel scenarios. For instance, Euroscore I was developed in 1999 using 19,030 patients collected over three months (September–December 1995) from 132 cardiac centres in eight countries [13]. Modifications to risk factors over time may have an resulted in its lack of discrimination and calibration compared to its successor score EuroSCORE (ES) II, developed in 2011.

5. Ethical and Fairness Concerns: Drift in the dataset may exacerbate problems with ethics or fairness. A system may reinforce preexisting biases and unfairly target particular groups if it was trained on biased or out-of-date data.

6. Regulatory Compliance: Using historical or drifted data could result in non-compliance with changing standards that need accurate, current information in regulated industries like healthcare.

The aim of this study was to investigate performance drift in existing ML models that have been used in prior cardiac surgery risk prediction research. The objectives were to (i) rank and assess the extent of performance drift in such cardiac surgery risk ML models over time; (ii) investigate any potential influence of dataset drift and variable importance drift on performance drift.

We have also added an extensive *Related Work* section in the *Introduction* section.

Future Work

Future studies shall also delve deeper into the relationships of the studied drift types with concept drift in cardiac surgery risk prediction.

The above important points have been incorporated in lines 123-186, 194-217, and 667-668.

*2. The rationale for introducing CEM as the primary performance metric, calculated as the geometric mean of 5 distinct individual metrics, is debatable and lacks strong justification. Although the geometric mean is less sensitive to outliers compared to the arithmetic mean, it raises the fundamental question of why these metrics need to be summarized. Is it merely to obtain a single quantitative measure for analysis, or does it aim to provide a more comprehensive understanding of overall model performance? It appears to serve primarily the former purpose, which may not be an appropriate practice given that the 5 metrics assess entirely different aspects of model performance: 1 – ECE for calibration, AUC for discrimination, 1 – Brier score (which already encompasses calibration and discrimination components), F_1_-score for threshold-specific discrimination, and net benefit index for cost-effectiveness. Consequently, interpreting the exact meaning of CEM becomes challenging, as it reduces these diverse aspects to a single numerical value. Therefore, I suggest just reporting and examining all 5 metrics individually, with or without highlighting certain ones as primary areas of interest.*

Response: Thank you for your comment. In our previous study, we found that combining the metrics covering all 4 aspects of discrimination, calibration, clinical usefulness, and overall accuracy into a single CEM improved the efficiency of cognitive decision-making (according to the Miller Law [4]) for selecting the optimal ensemble models [5,6]. This approach is useful for providing a consensus metric that enables models to be ranked in scenarios where, for example, 1 model could outperform another using 1 metric but underperform under a different metric. Furthermore, we demonstrated that such a consensus metric could be combined with drill-down analysis to further interpret the models using individual metrics [5]. Although AUC evaluates the diagnostic or predictive performance of the model, it does not directly reflect patient benefit. This is why we included a suit of other metrics, including the decision curve analysis net benefit index, that were found to be clinically pertinent from our prior study [7].

The above important points have been incorporated in lines 195-205.

*3. The manuscript used several statistical tests, and some of them are relatively less commonly used. Please provide a more detailed description of the objectives and specific statistical situations for each test used. Additionally, for the baseline nontemporal performance comparison, a more conventional approach for comparing AUC would be the use of the DeLong method (you could choose the best model as the reference), and bootstrapping can be used to assess the statistical significance when comparing other metrics.*

Response: Thank you. A list of statistical methods used for analyzing drift has been provided in Table 1 (please see the manuscript for the formatted version, thanks).

Objective Statistical Tests General Statistical Situations Rationale for Choosing Test Assumptions Checked

Non-temporal comparison of models Repeated measures One-Way ANOVA Comparison of multiple groups for differences Used for comparing means across multiple models Outliers (ANOVA assumptions), Normality (Shapiro-Wilk test)

Paired *t*-tests (Bonferroni Corrected) Comparison of paired observations between models To compare specific model pairs

Dunnett’s Correction Control for multiple comparisons Controls Type I error rate in comparing multiple treatments to a control group in one-way ANOVA

Analysis within specific time frames Kruskal-Wallis Test Comparison of multiple groups for differences (non-parametric) Non-parametric alternative for ANOVA in specific time frames Outliers (ANOVA assumptions), Normality (Shapiro-Wilk test)

Bonferroni Corrected Paired samples Wilcoxon test (Wilcoxon signed-rank test) Comparison of paired observations within time frames Non-parametric comparison of paired samples within time frames with control for Type I error rate in comparing multiple treatments

Dunn’s test Multiple pairwise comparisons within non-parametric groups Post hoc test for pairwise comparisons after Kruskal-Wallis test; Determines the magnitude of difference effects within time frames

Analysis between first 3 months of 2017 and 2019 Kruskal-Wallis Test Comparison of multiple groups for differences (non-parametric) Non-parametric comparison between time frames Outliers (ANOVA assumptions), Normality (Kolmogorov-Smirnov Test)

Paired samples Wilcoxon test (Wilcoxon signed-rank test) Comparison of paired observations between time frames Non-parametric comparison of paired samples between time frames

Bonferroni adjusted Dunn’s test Multiple pairwise comparisons between time frames Post hoc test for pairwise comparisons after significant Kruskal-Wallis results; Determines the magnitude of difference effects between time frames; with control for Type I error rate in comparing multiple treatments

Normality (Kolmogorov-Smirnov Test)

Analysis of discrimination, calibration, clinical utility, and overall accuracy drift Linear regression (with residual analysis) Assessing relationships and regression parameters To analyze linear relationships and model residuals Normality through histograms and QQ plots,

Seasonal Kendall Test (Non-parametric alternative if assumptions not met) Assessing association or trends when assumptions are not met Non-parametric test for assessing associations without assumptions Homoscedasticity through scale-location plots

We appreciate the suggestion regarding DeLong’s method for assessing AUC comparison. Future study could investigate the utility of DeLong’s method in measuring AUC differences, particularly in studies focusing on pairwise model comparisons. The computational demands of this strategy, which can be burdensome on large datasets, impacted our decision not to use it in the current study. However, given its proven importance in AUC comparisons, future studies with a focus on AUC evaluation and resource availability for controlling computational demands may explore using DeLong’s method. This method could aid in the refinement of comparison analyses in predictive modelling research by allowing for a more complete knowledge of AUC differences between models.

We wanted to analyse model performance across multiple metrics across time in this study. Although DeLong’s approach is often used for pairwise comparisons of the area under the curve (AUC), we chose not to utilise it due to its high computational demands [58], particularly on the large datasets present in this study. We chose a more comprehensive approach to capture the dynamics of model performance since this study included a broad examination across various performance metrics rather than focusing solely on AUC.

The above important points have been incorporated in lines 308-309, 580-586, and 659-665.

*4. During the training and internal validation phase with 5-fold cross-validation, additional details are needed to understand how the final model for each model type was selected for subsequent temporal validation, including whether hyperparameter tuning was carried out and whether there was a final refitting process on the entire training data set following the cross-validation, etc.*

Response: Thank you. Internal validation was performed using 5-fold cross-validation on the training and validation data set (2012-2016) to select model parameters. The final models were determined by retraining the models on the combined training and validation data set using the selected model parameters. Temporal validation was performed using the final models on the holdout data set (2017-2019) [8]. Further details on model development can be found in the *Model Specification* section in Multimedia Appendix 1.

Supplementary Section 2: Model Specification

Neural Network (Neuronetwork) was trained using 1000 epochs, with batch size of 20,000. The 2012-2016 dataset was split 70:30, with 70% used as training data and 30% as validation data for early stopping to reduce likelihood of overfitting [1]. The best model was saved using early stopping to prevent overfitting [2]. Binary cross-entropy loss was used as the loss function, with Adam as the optimizer [3], monitoring on accuracy as the metric. The final model configuration used for evaluation was the optimal set derived from the NACSA Bristol cohort from our previous study: input layer n=18 nodes, hidden layer one n=90 nodes, hidden layer two n=36 nodes and output layer one node [4].

3-fold Grid Search Cross Validation was applied for Weighted SVM and Xgboost using 2012-2016 dataset to determine the optimal hyperparameters to apply to 2017-2019 test dataset [5]. For Random Forest, we manually tuned parameters in response to model discrimination (AUC) evaluated with cross-validation (estimators n=700, maximum depth n=10, minimum samples split n=5, minimum samples leaf n=20) [4]. The ES II risk factors were fitted with an LR (retrained LR) model with Inverse of regularization strength (C) set to 1 [4].

The above important points have been incorporated in lines 258-261.

*5. The Introduction section should incorporate more background information on previous studies reporting or relating to performance variation in prediction models for cardiac surgery outcomes. In the Discussion section, it is also important to discuss how this work contributes to existing evidence in the context of these previous studies. Some relevant studies, based on my preliminary search, include Benedetto et al [9], Zeng et al [10], Mori et al [11], and potentially more.*

Response: Thank you. We have now included a *Related Works* section to enhance the *Introduction* section.

Related Work

In our previous study, we found that combining the metrics covering all four aspects of discrimination, calibration, clinical usefulness and overall accuracy into a single CEM improved the efficiency of cognitive decision-making (according to Miller’s Law [16] for selecting the optimal ensemble models [13,17]. This approach is useful for providing a consensus metric that enables models to be ranked in scenarios where for example one model could outperform another using one metric, but underperform under a different metric. Furthermore, we demonstrated that such a consensus metric could be combined with drill-down analysis to further interpret the models using individual metrics [13]. While AUC does evaluate diagnostic or predictive performance of the model, it does not directly reflect patient benefit. This is why we had included a suit of other metrics including the Decision Curve net benefit index that were found to be clinically pertinent from our prior study [18].

In our previous work [19], we had studied the calibration changes across two different time intervals using the calibration belt (overall external calibration) and Hosmer–Lemeshow goodness of fit *χ*^2^ statistics (calibration drift) approach within a single United Kingdom based hospital. A recent study extended our work to a Chinese national registry, Sino (Chinese) System for Coronary artery bypass grafting (CABG) Operative Risk Evaluation II (SinoSCORE II), using an set of ML models included lightGBM, CatBoost and a combination of variable selection approaches including Optuna for stepwise regression (SWR), BorutaSHAP (BS), and feature importance (FI) ranking [20]. Another study in the United States (U.S) had also investigated the calibration performance difference between Xgboost and Logistic Regression models built for the CABG patient cohort through pre-operative, intra-operative and combined variable sets from the Society of Thoracic Surgeons (STS) Adult Cardiac Surgery Database (ACSD) [21].

We have discussed these in the *Discussion* section where appropriate.

Discussion

Our previous study [19], while not involving the assessment of xgboost had also shown that calibration drift of Logistic Regression was less than that of Random Forest, while EuroSCORE I, Naïve Bayes and Neural Network performed poorly in terms of calibration. A recent study extending upon our work had shown that temporal and spatial calibration drift (comparison across regions and hosptials) to be severe across a range of ML models using a national Chinese registry [20]. In accordance with our view, the study highlighted that “future efforts may need to shift more towards enhancing model calibration robustness or recalibration for greater practical value” and that inclusion of intra-operative variables may be important to enhancing model performance. This Society of Thoracic Surgeons (STS) Adult Cardiac Surgery Database (ACSD) study [21], had shown that the inclusion of intra-operative variables improved both the discrimination and calibration performance of Xgboost and Logistic Regression models in CABG patients from the U.S.

The above important points have been incorporated in lines 195-217 and 560-572.

*6. Although the authors observed numerical declines in CEM and other metrics, the magnitude of these declines appears to be relatively small, particularly when considering metrics such as AUC. As a result, it is essential to discuss how to interpret this magnitude of drift in the context of clinical practice. In other words, what is the clinical significance of this variation in performance, and how does it justify the necessity of actively monitoring model drift in terms of cost-effectiveness? Please discuss.*

Response: Thank you for this interesting suggestion. We have now expanded the *Discussion* section to further discuss this suggested topic.

Discussion

Although the reported decreases in measures such as CEM and AUC may appear small, such changes are likely to impact the potential usage of ML models within clinical scenarios. If such models are to be used clinically for making decisions about the patient, even small changes in these metrics (which have been previously discussed[18] to be important in the cardiac surgery ML performance) can have an influence on risk assessment and patient outcomes, necessitating constant model drift monitoring. Prior research has shown that improving model calibration robustness or recalibration is necessary for practical value and that the “the significant decline in performance of previously established models in this study calls for continuing model updates”[20]. It is envisaged that collaboration between physicians and ML scientists is critical. Before mandating model updates, it is critical to establish metric-specific thresholds for acceptable reductions. A consensus approach, extensive experience in this area or a meta-analysis of current literature may be required for this collaborative decision-making process.

The above important points have been incorporated in lines 612-624.

*7. The conclusion should only focus on the primary findings outlined in the aims of the Introduction section. Avoid incorporating less central findings and speculative elements. Additionally, it may not be fair to suggest replacing the EuroSCORE II model simply based on the inferior performance in this study, since it was already established and this study essentially conducted an external validation for it, whereas the other machine learning models were developed using these data sets.*

Response: Thank you for this valuable suggestion. We have now revised the *Conclusion* section to make this more coherent and focused.

Conclusion

This study found that performance drift of ML and ES II over time could be explained through dataset drift patterns in cardiac surgery risk prediction. It was also found that variable importance drift could help to explain performance drift and support detection of dataset drift in the assessed models. The strong evidence of all models showing a decrease in at least 3 of the 5 individual metrics within CEM demonstrates the potential need to update the models over time but future work are required to determine suitable thresholds for mandating an update. Future work will be required to determine the interplay between Xgboost and RF, which have demonstrated less drift over time, and whether combining these through additional ensemble modelling could take advantage of their respective performance advantages.

The above important points have been incorporated in lines 671-690 and 534-535.

##### Minor Comments

*1. More detailed definitions and explanations should be provided for each performance metric.*

Response: Thank you for this helpful comment. This has now been included as part of the *Related Work* section.

Related Work

In our previous study, we found that combining the metrics covering all four aspects of discrimination, calibration, clinical usefulness and overall accuracy into a single CEM improved the efficiency of cognitive decision-making (according to Miller’s Law[16]) for selecting the optimal ensemble models [13,17]. This approach is useful for providing a consensus metric that enables models to be ranked in scenarios where for example one model could outperform another using one metric, but underperform under a different metric. Furthermore, we demonstrated that such a consensus metric could be combined with drill-down analysis to further interpret the models using individual metrics [13]. While AUC does evaluate diagnostic or predictive performance of the model, it does not directly reflect patient benefit. This is why we had included a suit of other metrics including the Decision Curve net benefit index that were found to be clinically pertinent from our prior study [18].

The above important points have been incorporated in lines 195-205.

*2. In the Methods section, please provide a clear outline of the inclusion and exclusion criteria. Additionally, consider including a flowchart that illustrates the data set development process, outlining how these criteria were applied.*

Response: Thank you. We have now updated the *Methods* section and Multimedia Appendix 1.

Methods

227,087 adults patients undergoing cardiac surgery between January 1, 2012 and March 31, 2019 were included. Congenital, transplant and mechanical support device insertion cases were excluded. A patient flow consort diagram is shown in Supplemental materials, Figure S1.

Supplementary Materials

Consort Diagram

Figure S1.1 Consort diagram showing flow of participants through the study.

The above important points have been incorporated in lines 224-227 and Figure S1 in Multimedia Appendix 1.

*3. I had difficulty understanding what “outliers” and “distribution” meant in the Results section for the baseline nontemporal performance of each model. I thought that each metric of each model should be just a numerical value and a 95% CI from bootstrapping.*

Response: Thank you for this helpful suggestion. We have now improved clarity of the *Results* section (see underlined parts below).

Baseline non-temporal performance

No extreme outliers were found when testing for ANOVA assumptions. The CEM scores from 1000 bootstraps were normally distributed for all three models except Xgboost,

The above important points have been incorporated in lines 368-369.

*4. The title of the manuscript should be an objective reflection of the overall study design and aim, rather than drawing conclusions from the findings.*

Response: Thank you for the valuable suggestion. We have now amended the title to “An assessment of performance drift in Machine Learning models for cardiac surgery risk prediction.”

The above important points have been incorporated in lines 2-4.

*5. I did not find the supplementary materials in the review system. I am not sure whether this issue is on my end or not.*

Response: Thank you. We have now reuploaded the latest changes in the supplementary materials to the journal upload page. We have also included updated figures within the supplementary materials with our responses wherever possible.

Thank you for your expert and invaluable review; this has been really thought provoking and had made significant contributions to improving the journal’s quality of output. We hope our changes will be met with your approval.

## Round 2 Review

### Reviewer CL

#### General Comments

*I appreciate the opportunity to rereview this manuscript. The authors’ efforts in revising their manuscript in response to previous concerns are commendable. This manuscript has been improved and is now in principle publishable. It could potentially be accepted upon reasonable response to a few follow-up minor comments, outlined below.*

#### Specific Comments

*1. About my previous major comment 1, the authors meticulously elaborated on (1) the reasons for performance drift and (2) its importance, which are both valid points. However, the current Introduction (lines 121-179) is quite lengthy. I recommend consolidating these 2 parts into a single paragraph, listing each point without the need for detailed individual explanations. Additionally, my query about the exact meaning of “the relationship between and within variable importance drift, performance drift, and actual dataset drift” remains unaddressed. Even though it was removed from the Introduction, it still appears in the abstract. I suggest the authors explicitly explain it to readers and incorporate it into the manuscript when first mentioned.*

Response: Please note that all line numbers refer to the marked version of the manuscript with tracked changes, uploaded as a supplementary file.

Thank you for this helpful suggestion. We have now improved the *Introduction* section by making it more concise and explaining the meaning of “the relationship between and within variable importance drift, performance drift, and actual dataset drift” as per your recommendations:

Introduction

In machine learning (ML), performance drift refers to the gradual loss in model performance caused by changes that call into question the model’s training assumptions. Key causes of performance drift include dataset drift, which refers to changes in the distribution of data between training and evaluation sets; variable importance drift, which involves changes in the significance of model variables; and calibration drift, which is characterised by decreased reliability in estimated probabilities. These factors can interact, as seen in a study of non-cardiac surgery [54]. Understanding the complex relationship between variable importance drift, performance drift, and dataset drift is important. This relationship explains how changes in the importance of specific variables, combined with changes in the actual data distribution, collectively influence the model’s overall accuracy and reliability as it performs over time. The wider implications are also significant, influencing decision-making, insight accuracy, generalisation [13], ethical considerations, and regulatory compliance across industries.

The above important points have been incorporated in lines 121-192.

*2. Regarding the justification for the CEM, the authors have added more explanation and supporting literature for its use. However, it would strengthen their case if they could provide examples from external studies or use cases where a similar practice (averaging different aspects of metrics for model performance evaluation) was used, beyond their own studies.*

Response: Thank you for your comment. We have now strengthened the case by providing examples of additional external studies where a similar practice have been applied:

Methods

The consensus approach for combining different metrics has previously been applied in a study on Covid-19 prediction [33]. In addition, this approach is similar to the simple additive weighting (SAW) multi-criteria evaluation approach for making a decision through the ranking of a set of competing criterions [34].

The above important points have been incorporated in lines 294-298.

*3. About the statistical tests for comparing AUC with the DeLong method, I believe that performing the DeLong test for AUC comparison is not overly computationally demanding, even on a relatively large data set. I recommend the authors explore commonly used R packages (eg, “pROC”) that facilitate AUC calculation and comparison with the DeLong method. The DeLong comparison typically requires paired variables of the label and 2 models’ predicted probabilities, and the 95% CI and P value are automatically calculated by bootstrapping these paired samples, which is relatively efficient.*

Response: Thank you for further explaining. We have know included the DeLong test in the baseline nontemporal comparison as you advised.

Baseline non-temporal performance (methods)

The Delong’s test was applied for determining whether there was a statistically significant difference across the AUCs of ROC curves for the top two best performing models.

Baseline non-temporal performance (results)

AUC performance was best for Xgboost (0.834) and RF (0.835), with the Delong’s test showing no statistically significant difference (*P*>.05).

Table below has been updated to include the Delong’s test:

Table 1a. Summary of statistical methods used for assessing drift.

Objective Statistical Tests General Statistical Situations Rationale for Choosing Test Assumptions Checked

Non-temporal comparison of models Repeated measures One-Way ANOVA Comparison of multiple groups for differences Used for comparing means across multiple models Outliers (ANOVA assumptions), Normality (Shapiro-Wilk test)

Paired *t*-tests (Bonferroni Corrected) Comparison of paired observations between models To compare specific model pairs simultaneously

Dunnett’s Correction Control for multiple comparisons Controls Type I error rate in comparing multiple treatments to a control group in one-way ANOVA

Delong’s test Comparison of the AUC of two correlated ROC curves To compare AUC of two models/tests during sensitivity testing

The above important points have been incorporated in lines 316-318 and 398-399 and Table 1.

*4. Regarding model tuning and specification of the best models (PS: I still cannot find the supplements, only a revised clean manuscript; I am not sure if this was due to issues from my end), I am curious why different tuning practices were used for different models, especially grid search for XGBoost and SVM but manual tuning for random forest.*

Response: Thank you for your helpful comment. For random forest, the final model configuration used for evaluation was the optimal set derived from our previous study on the NACSA Bristol cohort [9]. For the new models SVM and XGBoost, for which optimal parameters have not been investigated in our previous study [9], we applied 3-fold grid search cross-validation to determine the optimal hyperparameters.

The above important points have been incorporated by updating the *Model Specification* section in Multimedia Appendix 1.

*5. In response to the query about the clinical significance of the relatively small scale of performance drift, the authors referred to one of their previous studies briefly discussing this matter. However, it would be much clearer if the authors could more explicitly elaborate in this study and, if possible, provide additional analysis to support this argument.*

Response: Thank you for this invaluable comment.

Net benefit projection (methods)

To further understand the clinical significance of the performance drift over time, the fitted linear regression model intercepts and slopes was used to extrapolate the net benefit up to January 2030 for Xgboost and Neural Network models.

Net benefit projection (results)

To further understand the clinical significance of the performance drift over time, Figure 5 illustrates the expected net benefit decrease for a NN model and an XGBoost model. The blue line depicts the actual net benefit drop depending on the NN’s slope, transitioning to the projected red line using after March 2019. The green line represents the actual net benefit drop for the XGBoost model up to March 2019, changing to the projected purple line after March 2019. A clinical significant decrease (0.9035 to 0.8808) is shown for NN but not for Xgboost (0.9051 to 0.8962).

Figure 5. The actual and projected net benefit drift for NN and XGBoost models over time.

Discussion

However, through projecting the net benefit into the year 2030 based on the fitted linear regression, the decreases in the net benefit for Xgboost over time was shown to be clinically insignificant. On the contrary, the Neural network model showed a clinically significant drop in net benefit.

The above important points have been incorporated in lines 372-375, 548-557, and 641-644.

Thank you again for your expert and invaluable review; this has been really thought provoking and has made significant contributions to improving the journal’s quality of output. Thanks.

We hope this now addresses all your queries. Thank you.

## Abbreviations

AUC: area under the curve
CEM: clinical effectiveness metric
ECE: expected calibration error
EuroSCORE: European System for Cardiac Operative Risk Evaluation
NACSA: National Adult Cardiac Surgery Audit
SVM: support vector machine
XGBoost: extreme gradient boosting
